# Detection of *Toxoplasma gondii* in Acute and Chronic Phases of Infection in Immunocompromised Patients and Pregnant Women with Real-time PCR Assay Using TaqMan Fluorescent Probe

**Published:** 2018

**Authors:** Parisa MOUSAVI, Hossein MIRHENDI, Mehdi MOHEBALI, Saeedeh SHOJAEE, Hossein KESHAVARZ VALIAN, Shirzad FALLAHI, Setareh MAMISHI

**Affiliations:** 1. Dept. of Medical Parasitology and Mycology, School of Public Health, Tehran University of Medical Sciences, Tehran, Iran; 2. Dept. of Medical Parasitology and Mycology, School of Medicine, Isfahan University of Medical Sciences, Isfahan, Iran; 3. Center for Research of Endemic Parasites of Iran, Tehran University of Medical Sciences, Tehran, Iran; 4. Dept. of Medical Parasitology and Mycology, Faculty of Medicine, Lorestan University of Medical Sciences, Khorramabad, Iran; 5. Dept. of Infectious Diseases, Children Medical Center, Tehran University of Medical Sciences, Tehran, Iran

**Keywords:** Real time PCR, Immunocompromised patients, Pregnant women, RE gene, B1 gene, TaqMan fluorescent probe

## Abstract

**Background:**

*Toxoplasma gondii*, cause severe medical complications in infants and immune-compromised individuals. As using early, sensitive and rapid technique has major in diagnosis of toxoplasmosis, the present study was aimed to detect parasite by using from repetitive element (RE) and B1genes, in blood samples of seropositive immuno-compromised patients and pregnant women.

**Methods:**

A total of 110 peripheral blood samples were collected from seropositive cases with anti-*T. gondii* antibodies, including immunocompromised patients and pregnant women. DNA was extracted by a commercial kit and subjected to TaqMan probe-based real-time PCR assay by using primers and probes specific for RE and B1 genes, separately. The data were analyzed by Kappa test and SPSS-22 software.

**Results:**

In the pregnant women, 17 (68%) and 14 (56%) samples from 25 IgM+/ IgG+ cases and, 7 (25%) and 6 (21.4%) samples from 28 IgG+/IgM− cases were positive by RE and B1 real time PCR, respectively. Likewise, in immunocompromised group, 20 (66.6%) and 17 (56.6%) samples from 30 IgM+/ IgG+ cases and 2 (7.4%) and 2 (7.4%) samples from 27 IgG+/ IgM− cases were positive by RE and B1 real time PCR, respectively.

**Conclusion:**

Probe-based real time PCR assay is a quantitative approach for early diagnosis of *T. gondii* infection in clinical samples. Moreover, this method can be more appropriate in diagnosis of acute and reactivated toxoplasmosis. In addition our results indicated that RE gene is more sensitive than B1 gene.

## Introduction

*Toxoplasma gondii* is an obligate intra-cellular protozoan parasite with widespread distribution in the world that infects human and all warm-blooded animals (1, 2). Infection occurs by ingestion of oocysts that release from infected cats or consumption of raw meat containing the parasite cysts. Human infection is frequent and it is estimated that one-third of the human population are infected by *T. gondii* parasite (1). Although toxoplasmosis is often asymptomatic in immunocompetent individuals (3), it has been suggested as an important opportunistic infection in immunocompromised patients and pregnant women (4, 5). It can cause life-threatening infection and sever clinical signs such as ocular problem in immunocompromised patients, mental retardation and congenitally infection in children born from infected mother, and abortion in pregnant women (6, 7). Moreover, immune deficiency situation can lead to reactivation of latent infection and parasite proliferations that can result in sever complication such as cerebral toxoplasmosis and disseminated infection (8, 9). Early treatment of affected child would be possible if diagnosed quickly (10). Therefore, early diagnosis and treatment of infection in the primary stage is essential and may prevent the development of the disease.

Diagnosis of toxoplasmosis is usually achieved by detection of specific IgG and IgM antibodies to *T. gondii* (11). IgG avidity test is a confirmatory test for differentiate between acute and chronic phases of the infection but is not specific enough in immunocopromised patients (12, 13). Molecular techniques based on real-time quantitative PCR are sensitive tests for definitive diagnosis of the specific nucleic acid sequences of microorganism in different hosts with different samples (14). As molecular diagnosis is not depending on the immunological condition of the host, it would be ideal for immunocompromised patients. In recent years both conventional PCR and quantitative real-time PCR assays were developed for diagnosis of toxoplasmosis with high sensitivity (15, 16). Real time PCR can be used as an appropriate tool for sensitive and rapid detection of *T. gondii* in various clinical samples (17–20). Despite the previous studies related to these fields, there is not any standardization in molecular techniques in detection of *T. gondii* in acute and chronic phase of diseases. In current study, TaqMan real time PCR assays with a specific *Toxoplasma* probe targeting both RE and B1 targets are evaluated for quantitative detection of parasites in acute and chronic toxoplasmosis in seropositive blood specimen.

In this study two mentioned genes considered as multi copies and conserved marker in different strain of *T. gondii* parasite were selected to increase the diagnostic sensitivity (21).

## Materials and Methods

### Parasite preparation and DNA extraction

Tachyzoite forms of the virulent *T. gondii* strain RH, was prepared from the Laboratory of Toxoplasmosis, Tehran University of Medical Sciences, Tehran, Iran. 5 × 10^3^ tachyzoites in sterile PBS containing 100 IU/ml penicillin and 100 μg/ml streptomycin were injected intraperitoneally in female BALB/c mice. After 3 to 5 days, when clinical signs were observed, tachyzoites were collected from peritoneal exudates and centrifuged for 3 min at 3500 g, washed three times with 2ml cold PBS (pH 7.2) (22). The pellet resuspended in cold PBS and the parasites were counted by hemocytometer under a light microscope. DNA was extracted from purified parasite using commercial DNA extraction kit ((high pure PCR template preparation kit, Roche, Germany) according to the manufacturer′s instructions. Additionally, genomic DNA from indicated amounts of *T. gondii* tachyzoites (RH strain) was used as positive control in the PCR methods.

### Clinical samples

During August 2016 to July 2017, 110 blood samples were collected from two group including 55 seropositive blood samples with anti-*T. gondii* IgM and IgG antibodies (25 from pregnant women and 30 from immunocompromised patients) and 55 with anti-*T. gondii* IgG antibodies (28 from pregnant women and 27 from immunocompromised patients). All immunocompromised cases were positive for HIV limited to patients with CD4 cell counts < 200 cells/mm^3^. Also 110 seronegative bloods for anti-*T. gondii* antibodies from 55 pregnant women and 55 immunocompromised patients were collected as negative controls. All cases admitted to the different medical centers of Tehran University of Medical Sciences, Tehran, Iran. From each case, 5 ml peripheral bloods were collected for DNA extraction and serological tests.

### Enzyme immunoassays

Anti-*Toxoplasma* IgM and IgG antibodies were analyzed by VIDAS TOXO IgG and IgM Kit (bioMe′rieux, France). The assay combined an enzyme immunoassay method by immunocapture with a final fluorescent detection (ELFA). Analysis of the serum antibodies were assessed according to the kit manufactures. The test results were interpreted as follows: for IgM, < 0.55 IU/ml: negative, 0.55≤ to <0.65 IU/ml: equivocal, ≥ 0.65 IU/ml: positive and for IgG, < 4 IU/ml: negative, between 4≤ to <8: equivocal and ≥8: positive (23).

### DNA extraction from clinical samples

DNA was extracted from 110 seropositive cases from both groups (55 IgM +, IgG + and 55 IgM−, IgG +) and 110 seronegative cases as negative control using commercial extraction kit according to manufacturer's protocol (Roche, Germany). DNA was extracted from the whole blood samples and final DNA pellet was dissolved in 30 μl elution buffer for using in subsequent real time PCR reactions. As positive control, genomic DNA was extracted from 100 μl of tachyzoites of RH strain suspension containing 3×10^6^ parasites /ml.

### Real time PCR assay

The TaqMan probe-based real-time PCR assay was performed in this study. The reactions were done with an AB StepOne real-time PCR system (Applied Biosystems) in a final volume of 20 μl. After optimization, the reaction mixture contained 10 μl of RealQ Plus Master Mix for Probe (Ampliqon, Denmark), 0.4 μl of each primer (10 pmol/ μl), 0.4 μl of probe(10pmol/μl) labelled at the 5′ end with 6-carboxyfluorescein (FAM) and at the 3′ end with BHQ1 dye and 5 μl extracted DNA. The thermal cycling conditions included a 2 min at 50°C and 10 min at 95°C, amplification consisted of 45 cycles of 15 s of denaturation at 95°C, followed by 1 min of annealing and extension at 60°C. Each reaction was carried out in duplicate and the amplification runs contained *T. gondii* DNA as positive control and water and seronegative blood as negative controls. DNA extracted from given amounts of *T. gondii* tachyzoites (RH strain) were used as a positive control in each run. The nucleotide sequences of the primers and taqMan probes for both B1 gene and the RE repeated element are showed in Table 1.

**Table 1:** Nucleotide sequences of real time PCR primer/probe sets which targeted B1 and RE genes in this study

***Gene***	***Primer***	***Sequence (5′–3′)***	***Reference***
B1 gene	F primer	TCCCCTCTGCTGGCGAAAAGT	(Lin, et al. 2000)
	R primer	AGCGTTCGTGGTCAACTATC GATTG	
	TaqMan probe	FAM-CTGTGCAACTTTGGTGTATTCGCAG- BHQ1	
RE gene	F primer	CTTCGTCCAAGCCTCCGA	(Menotti, et al. 2010)
	R primer	GACGCTTTCCTCGTGGTGAT	
	TaqMan probe	FAM-CCCTCGCCCTCTTCTCCACTCTTCAA-BHQ1	

### Analytical sensitivity of real time PCR

To determine the limit of detection (LOD) value, five serial dilutions of *T. gondii* tachyzoite in human EDTA- blood were prepared ranging from 5000 to 1 tachyzoite and DNA was extracted from each dilution using a DNA extraction kit, according to the manufacturer’s protocol and real time PCR were carried out in duplicate. Results of the PCR (Ct values) were concluded by plotting the threshold cycle values against the numbers of tachyzoites per mL of blood (24).

### Statistical analysis

Data were analyzed using SPSS software (version 18; SPSS Inc., Chicago, IL) to identify statistically significant differences between observations the χ^2^ test was used and Cohen’s kappa calculates the agreement of serological and molecular tests.

### Ethical approval

This study was appraised and approved by the Ethics Committees of Tehran University of Medical Sciences, Iran (No: 32018).

## Results

### Analytical sensitivity of two different targets

**Fig. 1:**
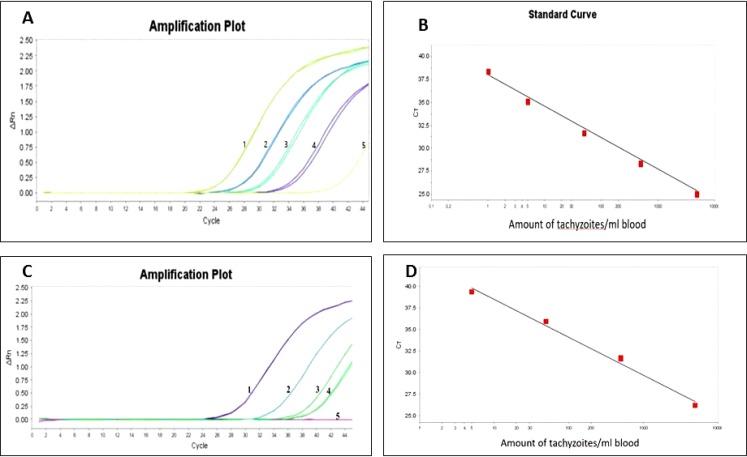
**A:** Real-time amplification plot with 5 serial dilutions ranged from 5000 to 1 tachyzoites as the initial DNA template based on RE target. **B:** Standard curve for 5 serial dilutions of *T. gondii* tachyzoites in human EDTA blood. CT values were plotted against amount of tachyzoites based on RE repeated element. **C:** Real-time amplification plot with 5 serial dilution ranges of 5000 to 1 tachyzoites as the initial DNA template based on B1 target. **D:** standard curve for 5 serial dilutions of *T. gondii* tachyzoites in human EDTA blood. CT values were plotted against amount of tachyzoites based on B1 gene. **(**Rn, fluorescent signal.**)**

Amplification plot with a CT value was obtained from described serial dilutions of *T. gondii* DNA in order to create a standard curve which were separately detected the RE and B1 gene at a concentration as low as 1 and 5 tachyzoites per ml of blood, respectively. The standard curve demonstrated a linear range across 5 serial dilution of *T. gondii* tachyzoites DNA with a correlation coefficient (R^2^) of 0.997 and 0.994 for RE and B1 respectively (Fig. 1) (Table 2).

**Table 2:** Threshold cycle (Ct) values for each dilution of *Toxoplasma* DNA tested in duplicate

***Tachyzoites***	***RE-CT min***	***CV %***	***B1-CT min***	***CV %***
5000	24.85±0.14	0.56	27.36±0.13	0.47
500	28.43±0.23	0.80	31.93±0.19	0.59
50	31.43±0.17	0.54	36.17±0.27	0.74
5	35.72±0.31	0.86	39.23±0.16	0.40
1	38.24±0.38	0.99	Undetected	-
NTC	Undetected	-	Undetected	-

NTC: Non Template Control

PCR accuracy was determined by estimate the mean coefficient of variation (CV) over each dilution point of the DNA tested in duplicate.

Results of real time PCR assay based on RE and B1 gene for detection of DNA in clinical samples are presented in Table 3. Following the real time PCR assay with both genes, all 110 seronegative samples were negative for toxoplasma DNA detection.

**Table 3:** Results of real time PCR assay for detection of *Toxoplasma gondii* DNA in acute and chronic phases of infection based on two target gene (each sample was tested in duplicate)

***Patients group***	***Patients category***	***RE- real time PCR***		***B1- real time PCR***	
		**Positive**	**CTs Rang**	**Positive**	**CTs rang**
Pregnant women	IgM+,IgG+ (25)	17	16–36	14	24–38
	IgM−,IgG+ (28)	7	20–37	6	24–38
	IgM−, IgG- (55)	0	-	0	-
ImmunocompromisedGroup	IgM+, IgG+ (30)	20	14–35	17	21–38
	IgM−,IgG + (27)	2	32–34	2	34–36
	IgM−, IgG- (55)	0	-	0	-

CT=threshold cycle

**Fig. 2:**
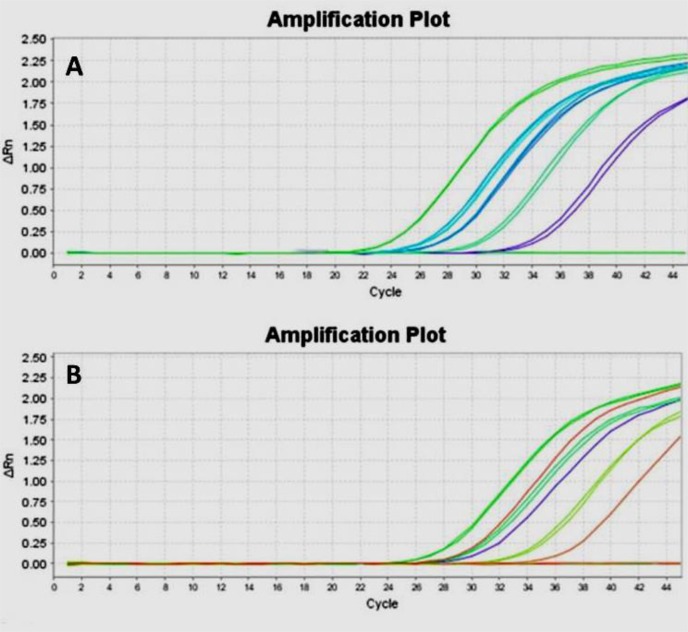
Real time PCR amplification plot for toxoplasmosis patients, positive and non-templet control based on RE (Image A) and B1 (Image B) genes

The results of duplicate RE and B1 probe based real time PCR assays on six randomly selected clinical samples are shown in Fig. 2. Among our positive result, 7 samples were positive by RE gene while with B1were negative (Table 3). Analysis of the samples by taqMan probe based real time PCR with two different gene targets (RE and B1) showed different detection sensitivity between two described genes.

## Discussion

In the recent years several studies have been conducted to diagnosing opportunistic infections in high risk hosts especially in immunocompromised patients and pregnant women (1). *T. gondii* is regarded as an important opportunistic pathogen parasite which can cause serious medical complications in susceptible hosts (7). Toxoplasmosis occurs in infants from an infected mother during pregnancy as a congenital infection (7) and in immunosuppressed individuals is often due to reactivation of latent infection, which presents neurological symptom and life threatening effects (25). Also acute acquired *T. gondii* infection in immunocompromised patients may occur and implicate several organs (26). Since serological detection of toxoplasmosis is not proper in patients who are immunocompromised, rapid and accurate molecular tools have developed to detection of infection in these cases especially when the parasite load is in low and in latent phase of infection (27). Additionally, in pregnant women early and definite diagnosis is essential to prevent the serious complications that need to high and rapid diagnostic tools.

Blood samples are convenient to obtain but due to limited amount of parasite DNA present in blood specimen, a sensitive tool and an appropriate gene target is very important to reach the best results (28, 29). Real time PCR assay was described as a high sensitive technique to detection of minute amounts of parasite DNA and produce the quantitative results in different types of samples (24, 25, 30–32). Additionally this method is appropriate for early and definite detection of infection in pregnant woman for well-timed treatment and to prevent severs complication.

Another aim of this study was to evaluate the sensitivity of the two target genes (RE and B1) for *Toxoplasma* detection in blood samples by real time PCR method. Both RE and B1, which has respectively 200–300 (33) and 35 copies (34) in *Toxoplasma* genome, reported to be conserve in different strain of *T. gondii* parasite and sensitive for molecular detection of toxoplasmosis (35). According to our results and based on the number of positive samples, CT value and standard curve, RE was more sensitive than B1 gene (*P*<0.05) and the analytical sensitivity of both RE and B1 real-time PCR assay described one and five tachyzoites per ml blood, respectively and the amplification was done in lower cycle of reaction based on RE target..

Rostami et al evaluated the frequency of *T. gondii* in HIV positive patients by ELISA and PCR and they reported that capture-ELISA and PCR could confirm the *T. gondii* acute infection in HIV positive patients, but suggested that more sensitive types of PCR are needed for precise diagnosis of acute toxoplasmosis in HIV positive patient (36).

The RE target is a highly conserved nucleotide sequence among the numerous strains and isolates of *T. gondii* using of sequencing technique (32). Multi-copy genes for diagnosis of *T. gondii* DNA are much more suitable and sensitive than single-copy gene (33). In a study, loop-mediated isothermal amplification (LAMP) and nested-PCR assay were applied to detection of *T. gondii* in blood samples of children with leukemia and compared the RE and B1 gene. Accordingly, RE and B1 targets present acceptable sensitivity, but RE was more sensitive (37). The B1 gene is extremely sensitive (0.05 parasite/reaction) and highly reproducible by using real time PCR and also been useful for the analysis of clinical samples, comprising blood and amniotic fluids (38). Fallahi et al compared the RE and B1 gene for detection of *T. gondii* infection in children with cancer and indicated that RE is more sensitive than B1 from blood samples (21).

In this study, positive results in peripheral blood samples were obtained in 46 and 39 Of 110 seropositive patient based on RE and B1 gene for both acute and latent phase of infection. This finding confirms that qPCR can identify parasites circulating in the peripheral blood of patients with acute and chronic phases of toxoplasmosis in immunocompromised patients and pregnant women, the CT values based on RE gene were less than B1 that showed the parasite DNA amplification in low cycle of reaction.

Additionally, based on the CT value from RE and B1 genes, the load of the parasites DNA in immunocompromised patients is more than pregnant women in acute phase of toxoplasmosis but in the chronic phase there is not any significant difference between two groups. The lack of easy access for people with AIDS and the difficulty in providing sample have been the limitations of the recent study. Based on our results, all seronegative patients were negative by real time PCR too. Most of patients with IgM+, IgG+ were positive by real time PCR and some patients with IgM−, IgG+ were positive that may be related to low parasites load in blood circulation in chronic phase of infection. In these cases the positive results may be the cause of the some reasons. This phenomenon may be for free DNA or because of the tattered old cyst. Because the immune system produces IgM antibodies in the first contact with the foreign antigen, but at a later stage, produced IgG antibodies forever (39).

## Conclusion

Probe based real time PCR assay as a sensitive diagnostic tool, provides an early and quantitative approach for molecular detection the acute and chronic phases of infection in clinical samples without any false positive result that may help to a targeted treatment. Also this method may be appropriate for screening of *T. gondii* infection in special cases such as immunocompromised group who usually fail to produce specific IgM or increased IgG titers. RE is more sensitive than B1 gene, and is recommended for routine diagnosis of toxoplasmosis.
